# DASFAA 20202 Special Issue Editorial

**DOI:** 10.1007/s41019-020-00143-z

**Published:** 2020-10-12

**Authors:** Yingxia Shao, Yanyan Shen, Bin Cui, Jeffrey Xu Yu

**Affiliations:** 1 Beijing University of, Posts and Telecommunications, Beijing, China; 2 Shanghai Jiao Tong University, Shanghai, China; 3 Peking University, Beijing, China; 4 The Chinese University of Hong Kong, Hong Kong, China

We are pleased to present a special issue of Data Science and Engineering (DSE), which contains a collection of papers from the DASFAA 2020 conference. Due to publication schedule, we publish four of these papers here, and postpone the publication of three additional ones to the coming DSE issues. In this issue, we also include three other papers selected from regularly submitted papers.

The International Conference on Database Systems for Advanced Applications (DASFAA) provides a leading international forum for discussing the latest research on database systems and advanced applications. The conference’s long history has established the event as the premier research conference in the database area. DASFAA 2020 focuses on research, development, and applications in relation to database, including a wide range of topics, such as neural network, knowledge graph, time series, social networks, attention mechanism, graph mining and crowdsourcing. DASFAA 2020 was held during September 24–27, 2020, in Jeju, Korea. The conference was originally scheduled for May 21–24, 2020, but postponed due to the outbreak of COVID-19 and its continual spreading all over the world. DASFAA 2020 attracted a total of 487 research paper submissions. The conference program committee selected 119 full research papers (acceptance ratio of 24.4%) and 23 short papers to be presented at the conference and published in the proceedings [1–3]. In addition, the committee included 4 industrial papers, 15 demo papers, and 3 tutorials in the program. Last but not least, to shed the light on the direction where the database field is headed to, the conference program included four invited keynote presentations by Amr El Abbadi (University of California, Santa Barbara, USA), Kian-Lee Tan (National University of Singapore, Singapore), Wolfgang Lehner (TU Dresden, Germany), and Sang Kyun Cha (Seoul National University, South Korea).

Seven extended papers were selected from among all the accepted papers by the special issue guest editors Yingxia Shao, Yanyan Shen, Bin Cui, and Jeffrey Xu Yu, based on the relevance to the journal and the reviews of the conference version of the papers. The authors were asked to revise the conference paper for journal publication and in accordance with customary practice of adding 30% new materials. The revised papers again went through the review process in accordance with DSE guidelines and are finally presented to the readers in the present form.

The seven extended papers cover a variety of topics related to database, data science and engineering. This issue includes four papers. The first “Exploiting Latent Semantic Subspaces to Derive Associations for Specific Pharmaceutical Semantics” presented an approach to extract interpretable latent semantic subspace from the neural-embedding models in the biomedical domain. The second paper “AGTR: Adversarial Generation of Target Review for Rating Prediction” introduced a recommendation model that can generate the unseen reviews for target users and items with adversarial training for rating prediction. The third paper “Show me the crowds! Revealing Cluster Structures through AMTICS” proposed a novel and efficient visualization approach to interactively and fast mine coarse insights in very dynamic and rapid changing applications. The fourth paper “Consensus-based Group Task Assignment with Social Impact in Spatial Crowdsourcing” proposed a novel framework for group task assignment based on two preference-related techniques—social impact-based preference modeling and preference-aware group task Assignment.

In upcoming issues of DSE, three additional papers selected from DASFAA 2020 will be published. The first “Parrot: A Progressive Analysis System on Large Text Collections” presented a sample-based progressive query processing model that is based on an incremental execution engine for large text collections. The second paper “Blocking techniques for Entity Linkage: a semantics-based approach” presented two automatic blocking strategies that capture the semantic properties of data by means of recent Deep Learning frameworks. The last paper “Heterogeneous CPU-GPU Epsilon Grid Joins: Static and Dynamic Work Partitioning Strategies” presented a heterogeneous CPU-GPU distance similarity join algorithm via efficiently partitioning the work between CPU and GPU.

We hope that the readers enjoy this special issue. We would like to acknowledge the work done by all the authors and their willingness to contribute their papers to this special issue. We thank all the reviewers for their expert comments and assistance in timely reviews. Finally, a note of thanks is to DSE Editors-in-Chief X. Sean Wang and Timos Sellis for their guidance and support in this process.
